# Metastatic adult neuroblastoma with spontaneous tumor lysis syndrome

**DOI:** 10.4322/acr.2020.181

**Published:** 2020-09-02

**Authors:** Tarsila Vieceli, Ana Laura Jardim Tavares, Renata Pibernat de Moraes, Gustavo Adolpho Moreira Faulhaber

**Affiliations:** a Hospital de Clinicas de Porto Alegre, Internal Medicine Department. Porto Alegre, RS, Brazil.

**Keywords:** neuroblastoma, adult neuroblastoma, tumor lysis syndrome

## Abstract

Neuroblastoma (NB) is a solid tumor of the sympathetic nervous system, most commonly found in childhood, standing for 7% of all pediatric malignancies. The incidence in adults is markedly smaller: 1 case per 10 million adults per year. We report the case of a previously healthy 27-year-old woman who started with lumbar pain, asthenia, and abdominal distension over the last month. A chest and abdomen tomography scan showed a huge mass in the upper left hemithorax and marked hepatomegaly. The diagnosis was confirmed by hepatic and lung biopsies. On day 4, after admission, the patient started chemotherapy. On the following days, she had severe vaginal bleeding, epistaxis, worsening of the hepatic function markers, refractory shock, and multiple organ dysfunction. She died on the twelfth day of admission. We also present a review of adult cases of NB reported in the past 5 years.

## INTRODUCTION

Neuroblastoma (NB) is a malignancy of the autonomic nervous system, originated in primitive sympathetic neural cells from the adrenal medulla and paraspinal sympathetic ganglia.^1^ It is frequently located in the adrenal medulla, neck, chest, and pelvis.^2^

NB is a relatively common malignancy in childhood, accounting for 7% of all pediatric malignancies.^3^ The highest incidence rate (55.2 per 1 million) occurs in children in the first year of life.^4^ The incidence of NB in adults is markedly smaller: 1 case per 10 million adults per year.^5^ Less than 100 reported cases of adult NB are reported in the literature.^6^ The most common primary sites for adults are retroperitoneum (70.3%), adrenal (37%), abdomen (33.3%), and thorax (18.5%).^7^

The age is an important factor in staging: children who are older than 18 months at diagnosis have a poorer prognosis.^8^ 5-year survival for infants diagnosed with early-stage NB approaches 90%, whereas adults have a 36.3% of 5-year survival rate.^9^ Therefore, all adults diagnosed with NB are considered with a high risk of death, regardless of the staging.^2^

## MATERIALS AND METHODS

We reviewed recent literature on neuroblastoma, including case reports published in the past 5 years available in Pubmed and Scielo. We have decided to exclude cases of olfactory neuroblastoma, considering that this type of NB has a different epidemiological distribution. A search was performed, including the term “adult neuroblastoma” under case reports published in Portuguese or English.

## RESULTS

The literature search yielded 148 results; 22 out of them were excluded because other tumors were reported, 18 were pediatric cases, 13 were cases of olfactory NB, 76 were not clinical case reports as such, and 4 had incomplete information regarding treatment and follow-up. Ultimately, 15 cases were included (Table 1).

**Table 1:** Summary of the cases of Adult Neuroblastoma reports

author	Gender	Age (y)	Clinical presentation	Treatment	Outcome
Bukhari et al.^10^	F	31	NB compressing spinal cord	Chemo = V, D, C	septic shock, death during chemotherapy
Naeem et al.^11^	M	29	Sacral NB associated to soft tissue mass extending into the central canal	Chemo Ca,E,C,D,Cy; RDT is scheduled	No recurrence at 8-month follow-up
Zhang and Feng^12^	F	75	NB limited to left adrenal gland	Surgery alone; patient decided not to receive chemo or RDT	Death 22 months after resection with bilateral lung and brain metastasis
Tan et al.^13^	F	46	NB under left dorsal pleura, no metastases	Surgery	No recurrence at 36-month follow-up
Yanik et al.^14^	M	40	NB of the superior vena cava and the subcarinal area with adjacent vascular and mediastinal invasion	Chemo I, Ca, E surgery was attempted with complete resection	No recurrence at 3-month follow-up
Martinez-Ciarpaglini et al.^15^	M	40	Inguinal NB	Chemo V, Act-D, C/I, E, plus RDT and surgery	No recurrence at 3-month follow-up
Ma et al.^16^	M	24	Mediastinal NB, relapsed in the retroperitoneum	Surgery	No recurrence at 12-month follow-up
Huang et al.^17^	F	41	Renal neuroblastoma, retrocaval lymphadenopathy and retroperitoneal metastasis	Surgery followed by Chemo Ci, D, E, C	No recurrence at 24-month follow-up
Wu et al.^18^	F	20	Retroperitoneal NB invading lateral abdominal wall, spine, left kidney, left kidney vein, splenic vein, pancreas, retroperitoneal tissue and left colon	Surgery, RDT, Chemo C, V, A and dimethyl triazeno imidazole carboxamide and James protocol	Recurrence in left clavicle, death 6 mo. after surgery
He et al.^19^	F	28	Cervix NB	Surgery, chemotherapy and RDT	No recurrence at 12-month follow-up
Yao et al.^20^	M	16	NB in the right temporal lobe and hippocampus	Surgery, temozolomide and RDT	No recurrence at 60-month follow-up
Bove et al.^21^	M	38	Parotid NB	Surgery	No recurrence at 36-month follow-up
Kałużna-Oleksy et al.^22^	F	39	Heart NB. NB in the spine treated at the age of 21	Not eligible for treatment. Pacemaker implanted	Death after metastases in the CNS, lungs and abdomen
Godkindi et al.^23^	M	24	Retroperitoneal ganglioneuroblastoma, metastasis to the left supraclavicular region	Chemo I, Ca, E, Surgery, RDT	No recurrence at 3-month follow-up
Wiesel et al.^24^	M	62	Thymic NB	Surgery, RDT, and immunosuppression	No recurrence at 6-month follow-up

Act-D= actinomycin-D; A= Adriamycin; CNS= central nervous system; C= cyclophosphamide; Ca= carboplatin; Ci= cisplatin; Cy= cytotecan; D= doxorubicin; E=etoposide; F= female; I= ifosfamide; M= male; NB= neuroblastoma; RDT= radiotherapy; V = vincristine; Y=year.

### Case Presentation

A previously healthy 27-year-old Brazilian woman presented to the ER complaining of lumbar pain, asthenia, and increased abdominal girth for the past month. She noticed a 5kg weight loss over the period and reported one episode of fever about a month before. The patient was recently diagnosed with anemia in a primary care facility and had received two red blood cell transfusions in the past month. She mentioned occasional episodes of epistaxis.

Her previous medical record was unremarkable; she took oral contraceptives as the only medication. She had no family history of malignancy.

On admission, the patient looked pale, frail, and had evident increased abdominal girth. Her heart rate was regular, 78 beats per minute, and her respiratory rate was within the normal range. Breath sounds were reduced in the upper left hemithorax. She had diffuse petechiae and moderate lower extremity edema. Her abdomen was distended and painful on palpation. A laboratory workup disclosed microcytic anemia (Hb 8.7g/dL). Total iron-binding capacity was 140ug/dL (normal range(NR) 135-392ug/dL), transferrin saturation was 37.2% (NR 25-50%) and ferritin was 1343ng/mL (NR 13-150ng/mL); normal white blood cell (WBC) count with left shift (8200/uL total WBC - 71% neutrophils, 10% lymphocytes, 4% monocytes, 1% blasts, 7% band neutrophils, 1% metamyelocytes, 4% myelocytes and 1% promyelocytes), thrombocytopenia (35 x 10^3^/dL), low serum albumin (2.9g/dL, NR 3.5-5.2g/dL), slightly increased International Normalized Ratio (1.27), high lactate dehydrogenase (LDH) (847U/L, NR 125-220u/L) and increased C-reactive protein (127mg/L, NR <5,0mg/L). Uric acid was slightly elevated (7.7mg/dL). Renal function, blood urea nitrogen (BUN), electrolytes, bilirubin levels, and hepatic transaminases were within the normal range. HIV, HCV, syphilis, and Hepatitis B virus serologies yielded negative results.

Bone marrow biopsy was performed, showing diminished cellularity and lymphocyte predominance (41%). Immunophenotyping yielded an increase in T-cells and a 2% non-hematopoietic cell population positive for CD56, CD81, CD9, and CD90; negative for CD45 and CD99.

**Figure 1:**
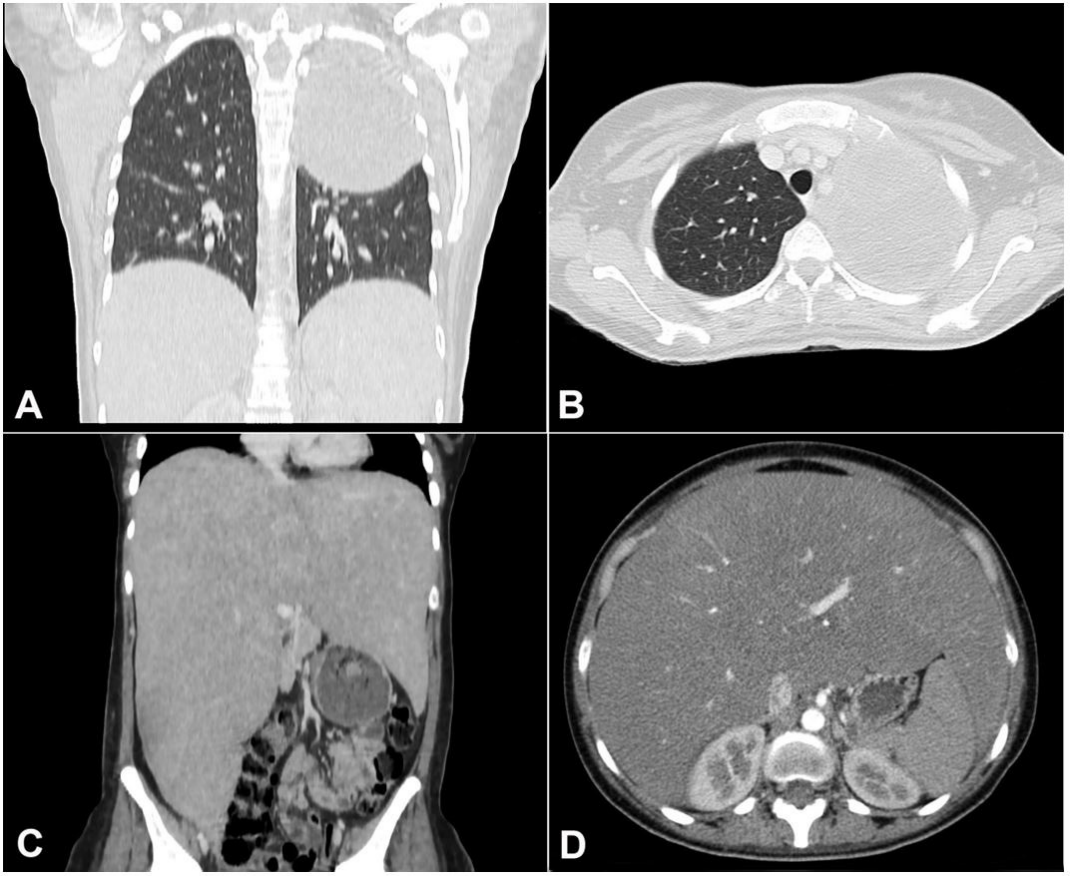
**A –** Chest CT scan Coronal plane – and **B –** axial plane -revealing a large mass in the left hemithorax; **C –** abdominal CT scan coronal plane and **D** axial plane - showing remarkable hepatomegaly.

A chest and abdomen CT scan showed a huge mass in the upper left hemithorax and dramatic hepatomegaly (Figure 1). The thoracic mass measured 10.7 x 10.3 x 9cm causing compression on the left pulmonary artery. There were no signs of mediastinal or bone invasion; thoracic lymph nodes were unremarkable. The liver was enlarged, measuring 35cm in the longitudinal axis. Both adrenal glands, kidneys, spleen, and pelvic organs had a normal configuration.

Ultrasonography-guided biopsy of the lung mass was attempted but failed to yield viable material for analysis. The patient was then submitted to a CT-guided biopsy of both lung and hepatic masses. During the subsequent day, she presented with tachypnea and dizziness. Laboratory workup of third day after admission revealed uric acid (13.1mg/dL, NR 2,6-6mg/dL), AST (904U/L, NR 5-34U/L), serum calcium (10.3mg/dL, NR 8.4-10.2mg/dL), phosphate levels (4.9 mg/dL, NR 2,3-4,7mg/dL), Potassium (5.4mEq/L, NR 3,5-5,1mEq/L, a 28% increase from baseline) and INR of 3.98. Despite receiving multiple red blood cell transfusions, her hemoglobin level remained persistently low (6.5g/dL). She also received platelet transfusions, intravenous vitamin K, fluid repletion, and allopurinol.

**Figure 2:**
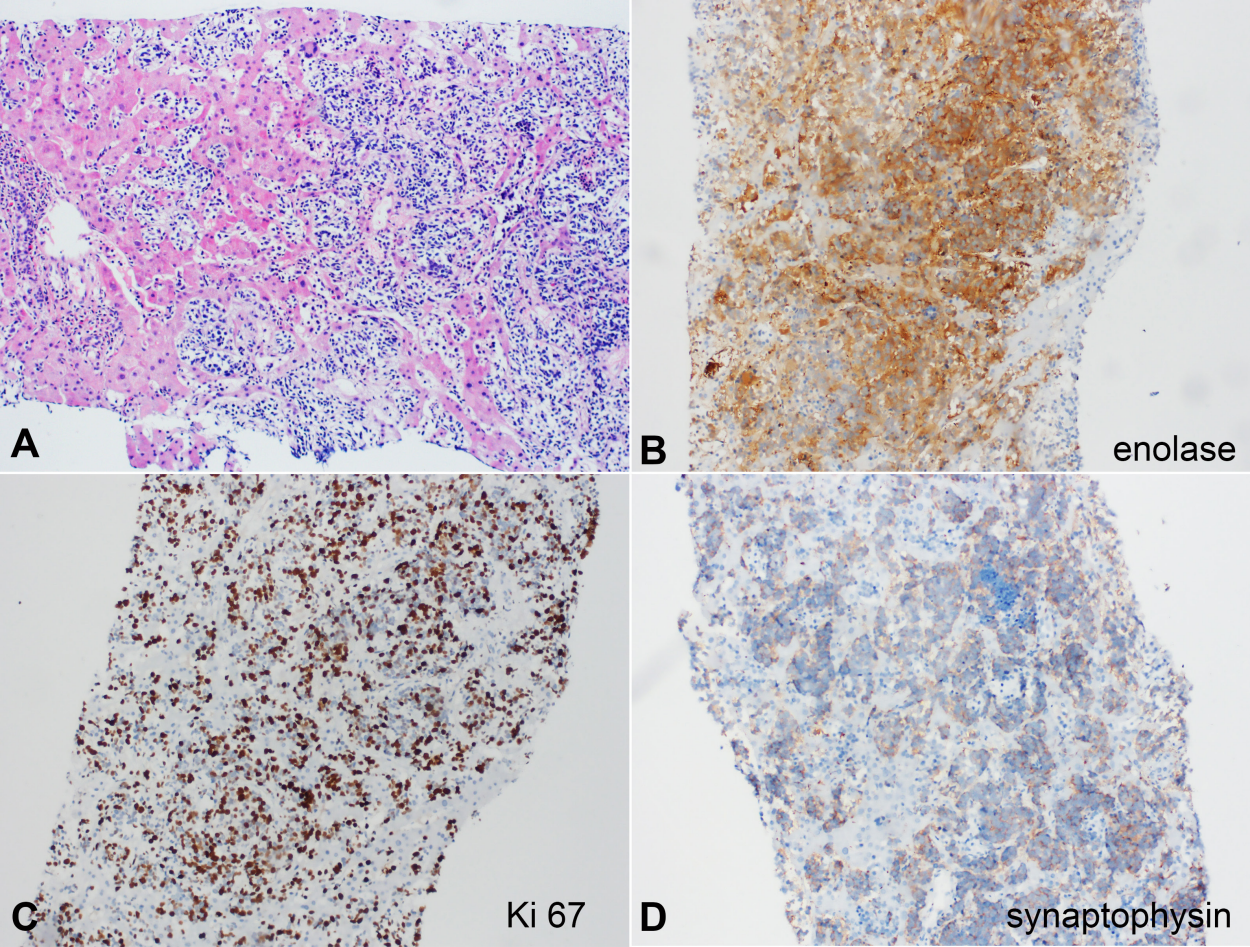
Photomicrographs of the liver biopsy. A - metatstatic neroblastoma in the liver parenchyma (H&E), B - enolase positive reaction, C - Ki 67, D - synaptophysin weak positive reaction.

Results for the hepatic and second lung biopsy revealed metastatic neuroblastoma (Figure 2). The immunohistochemistry (IHC) panel was weakly positive for synaptophysin (MRQ-40) and positive for enolase (MRQ-55) and Ki-67 in over 50% of the cell population.

**Figure 3:**
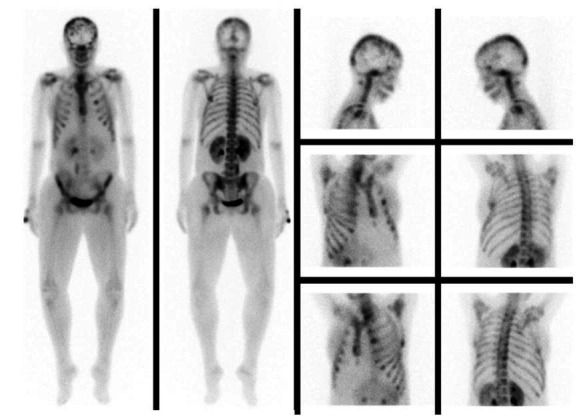
Bone scintigraphy with increased radiotracer uptake in the skull, third left costal arch, sternum, and T9.

Bone scintigraphy revealed an increased uptake in the skull, third left costal arch, sixth right costal arch, the lower third of the sternum, and in T9 (Figure 3).

**Figure 4:**
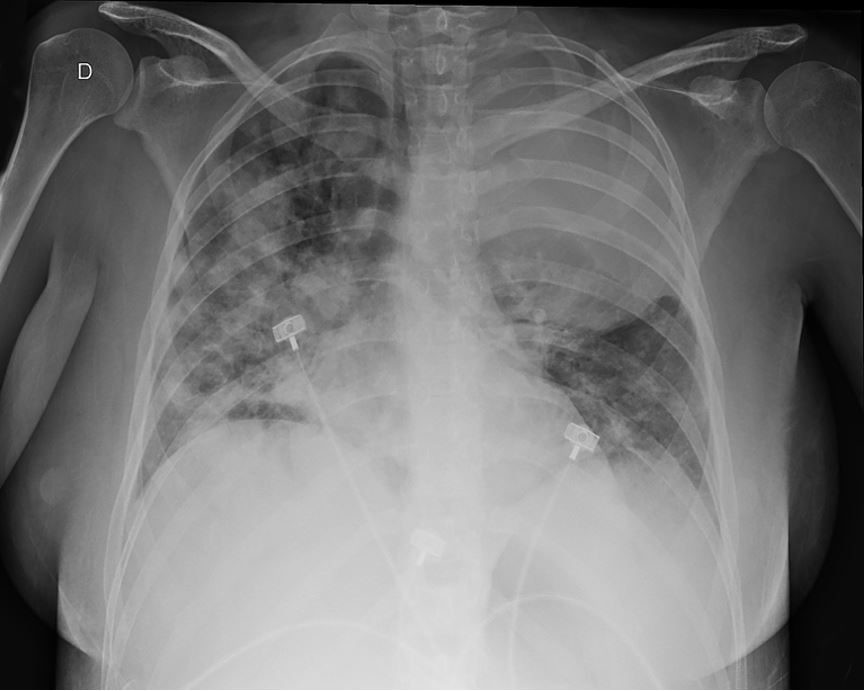
Chest radiography in day 4 of admission, prior to ICU admission, and subsequent orotracheal intubation. D stands for right side.

On day 4, after admission, the patient started receiving chemotherapy - cyclophosphamide (400mg/m^2^) and topotecan (1.2mg/m^2^). Epistaxis, respiratory, and neurologic status decreased dramatically in the following hours. She was submitted to orotracheal intubation and was admitted to the Intensive Care Unit (ICU). A chest X-ray prior to intubation revealed diffuse bilateral infiltrate (Figure 4).

On the following day, the renal function worsened (BUN 59.2mg/dL) and positive fluid balance. Her hemodynamic status rapidly deteriorated, requiring vasoactive drugs. She was submitted to continuous hemodialysis. Empirical antibiotic and antifungal therapy were prescribed.

During the following days, the patient presented with severe vaginal bleeding and epistaxis, despite administration of tranexamic acid and many platelets and plasma transfusions. She developed worsening hepatic function (AST 3860U/L; ALT 740U/L), increased lactate levels (8mmol/L - NR 0.5-1.6mmol/L), and refractory shock, resulting in multiple organ dysfunction. She died on the twelfth day of admission.

## DISCUSSION

No standard therapy for adults diagnosed with NB is a consensus yet, due to the scarcity of cases; high-dose of cytotoxic chemotherapy remains the treatment of choice. The most common regimens include cisplatin, cyclophosphamide, topotecan, etoposide, vincristine, and doxorubicin.^25^ ^,^ ^26^ A cohort study involving 118 adults with NB concluded that the treatment with chemotherapy, surgery, and radiation yielded better survival rates in patients with the L1 disease (disease restricted to the area of origin).^26^

In our literature review, cases of NB classified as L1 usually had a good prognosis, even those undergoing only surgical treatment. However, all patients who developed metastases died because of the tumor, implying that metastatic NB has a very poor prognosis. Our patient had a diffuse metastatic disease at diagnosis and rapidly evolved to spontaneous tumor lysis syndrome, which is also associated with a poorer prognosis. Although prognosis and survival rates are dismal for all adult patients with NB, the survival rates vary according to the stage. Adults with early-stage disease experience a 5-year overall survival rate of 90%, compared to 13-19% in adults with metastatic disease,^25^ ^,^ ^26^ which is present in about one-third of the patients at diagnosis.^7^

Podda et al.^7^ reviewed 27 adult patients submitted to a therapeutic protocol according to the disease stage: Stage I - surgery only; Stage II - surgery and postoperative radiotherapy; Stage III and IV - Chemotherapy and local therapy (surgery or radiotherapy) after the 6th course. In this study, the 5-year survival rate was 83% for stages I and II and 28% for stage III. All patients with stage IV disease relapsed and died due to the disease progression.

## CONCLUSION

We report a rare case of adult neuroblastoma presenting with spontaneous tumor lysis syndrome. In our review of recent literature, most cases with the limited disease had a good prognosis, and some had a good outcome with surgery alone.

As far as we know, we could not find any case report of NB presenting with spontaneous tumor lysis syndrome at diagnosis between 2015 and 2020.

## References

[1] Brodeur GM (2003). Neuroblastoma: biological insights into a clinical enigma. Nat Rev Cancer.

[2] Maris JM (2010). Recent advances in neuroblastoma. N Engl J Med.

[3] Becker J, Wilting J (2018). WNT signaling, the development of the sympathoadrenal-paraganglionic system and neuroblastoma. Cell Mol Life Sci.

[4] Gurney JG, Severson RK, Davis S, Robison LL (1995). Incidence of cancer in children in the United States. Sex-, race-, and 1-year age-specific rates by histologic type. Cancer.

[5] Siegel R, Naishadham D, Jemal A (2012). Cancer statistics, 2012. CA Cancer J Clin.

[6] Rogowitz E, Babiker HM, Kanaan M, Millius RA, Ringenberg QS, Bishop M (2014). Neuroblastoma of the elderly, an oncologist’s nightmare: case presentation, literature review and SEER database analysis. Exp Hematol Oncol.

[7] Podda MG, Luksch R, Polastri D (2010). Neuroblastoma in patients over 12 years old: a 20-year experience at the Istituto Nazionale Tumori of Milan. Tumori.

[8] Berthold F, Spix C, Kaatsch P, Lampert F (2017). Incidence, survival, and treatment of localized and metastatic neuroblastoma in Germany 1979-2015. Paediatr Drugs.

[9] Esiashvili N, Goodman M, Ward K, Marcus RB, Johnstone PA (2007). Neuroblastoma in adults: incidence and survival analysis based on SEER data. Pediatr Blood Cancer.

[10] Bukhari N, Harfouch B, Alotaibi MS, Al-Harbi H, Chamdine O (2020). Immediate response to chemotherapy in an adult neuroblastoma patient presenting with cord compression. Case Rep Neurol Med.

[11] Naeem M, Maluf H, Baker JC, Jennings JW (2019). Primary osseous sacral neuroblastoma in an adult. Skeletal Radiol.

[12] Zhang H, Feng Z (2019). Adrenal neuroblastoma in an elderly adult: a case report and review of the literature. J Med Case Rep.

[13] Tan YB, Li JF, Li WS, Yang RL (2019). Primary thoracic neuroblastoma in an adult: a rare case report. Medicine (Baltimore).

[14] Yanik F, Karamustafaoglu YA, Yoruk Y (2019). A rare mediastinal occurrence of neuroblastoma in an adult: case report. Sao Paulo Med J.

[15] Martinez-Ciarpaglini C, Machado I, Yoshida A (2019). Extra-adrenal adult neuroblastoma with aberrant germ cell marker expression: maturation after chemotherapy as an important clue to a challenging diagnosis. Int J Surg Pathol.

[16] Ma X, Yang Y, Wang Z (2018). Surgical removal of adult recurrent neuroblastoma located in the posterior mediastinum and retroperitoneum: a case report. Medicine (Baltimore).

[17] Huang MD, Hsu LS, Chuang HC (2018). Adult renal neuroblastoma: a case report and literature review. Medicine (Baltimore).

[18] Wu XL, Dai YJ, Sun GY (2018). Adult neuroblastoma in the retroperitoneum: a case report. Medicine (Baltimore).

[19] He Y, Yao M, Zhang X, Sun P, Gao H. (2017). A very rare adult case of cervical neuroblastoma. Int J Clin Exp Pathol.

[20] Yao PS, Chen GR, Shang-Guan HC (2017). Adult hippocampal ganglioneuroblastoma: case report and literature review. Medicine (Baltimore).

[21] Bove A, Percario R, De Carlo A, Zappacosta B (2017). A case report of the first ganglioneuroblastoma of the parotid gland of an the adult. Ann Ital Chir.

[22] Kałużna-Oleksy M, Wachowiak-Baszyńska H, Migaj J, Stefaniak S, Mróz M, Straburzyńska-Migaj E (2016). Unresectable heart neuroblastoma in an adult: a natural follow-up. Pol Arch Med Wewn.

[23] Godkhindi VM, Basade MM, Khan K, Thorat K (2016). Adult neuroblastoma-case report and literature review. J Clin Diagn Res.

[24] Wiesel O, Bhattacharyya S, Vaitkevicius H, Prasad S, McNamee C (2015). Ataxia induced by a thymic neuroblastoma in the elderly patient. World J Surg Oncol.

[25] Yalçin B, Kremer LC, van Dalen EC (2015). High-dose chemotherapy and autologous haematopoietic stem cell rescue for children with high-risk neuroblastoma. Cochrane Database Syst Rev.

[26] Conter HJ, Gopalakrishnan V, Ravi V, Ater JL, Patel S, Araujo DM (2014). Adult versus pediatric neuroblastoma: the M.D. Anderson Cancer Center Experience. Sarcoma.

